# Utilising a web-based e-learning platform for orthodontic case analysis training: a randomised controlled trial

**DOI:** 10.1186/s12909-026-09968-0

**Published:** 2026-07-24

**Authors:** Norbert Alexander Lang, Laura Bell, Lorena Backes, Franziska Alina Lang, Michael Wolf, Katharina Mücke, Teresa Kruse, Kathrin Becker, Isabel Knaup, Martin Lemos

**Affiliations:** 1 Department of Orthodontics and Dentofacial Orthopedics, Medical Faculty, RWTH Aachen University, Pauwelsstr. 30, Aachen, 52074 Germany; 2 Audiovisual Media Center, Medical Faculty, RWTH Aachen University, Aachen, Germany; 3 Department of Orthodontics and Dentofacial Orthopedics, Medical Faculty, Heinrich Heine University, Düsseldorf, Germany; 4 Department of Orthodontics, Faculty of Medicine and University Hospital Cologne, University of Cologne, Cologne, Germany; 5 Department of Orthodontics and Dentofacial Orthopedics, Medical Faculty, Charité, Berlin, Germany

**Keywords:** Technology-enhanced learning, Digital dental education, E-learning, Teaching innovation, Diagnostic analysis, Diagnostic reasoning

## Abstract

**Background:**

In orthodontic case analysis, structured clinical reasoning is required to achieve a diagnosis and establish a treatment decision. In this randomised controlled trial, a web-based training app was compared with conventional analogue case planning in undergraduate dental students.

**Methods:**

Twenty-eight undergraduate dental students from RWTH Aachen University were randomised to either a digital training group, using the web-based app Orthotrainer, or an analogue training group, performing conventional case analyses with physical models and printed diagnostic records. After a baseline knowledge and online readiness assessment, both groups completed two standardised training sessions. Following the sessions, the ease of use was assessed using the System Usability Scale and the User Experience Questionnaire. The final analogue module exam tested knowledge transfer. Additionally, 27 orthodontic educators from four university orthodontic departments were surveyed regarding analogue correction practices.

**Results:**

Baseline orthodontic knowledge was comparable between groups (analogue: M = 13.07 ± 3.15 vs digital: M = 12.86 ± 2.41, *p* = 0.842). Both groups demonstrated significant performance improvement across training sessions separated by three weeks, ß = 8.31, 95% CI [2.66, 13.96], *p* = 0.005, without overall group differences. Exploratory subarea analyses revealed analogue superiority in extraoral assessment (b = 23.37, 95% CI [7.31, 39.42], *p* < 0.001), digital advantage in radiographic evaluation (b = -17.34, 95% CI [-33.40, -1.29], *p* = 0.002) and no difference in the model analysis. There were no differences between the groups in the final module exam. The usability assessment yielded a mean SUS score of M = 78.89 ± 14.11 for digital training (compared to the analogue training, M = 40.30 ± 11.19, *p* < 0.001), while students rated the UEQ for the digital version higher in the dimensions of pragmatic, hedonic quality and overall quality (all *p* < 0.001). Among 27 surveyed educators, analogue correction was rated time-intensive, with preference for digitalisation.

**Conclusions:**

The Orthotrainer web application delivered diagnostic performance equivalent to that of conventional analogue training, proving particularly effective in radiographic analysis. Higher usability and user experience ratings support its integration into routine teaching. These findings suggest that structured digital tools can support the development of diagnostic reasoning and serve as a valuable addition to traditional orthodontic education.

**Supplementary Information:**

The online version contains supplementary material available at 10.1186/s12909-026-09968-0.

## Background and rationale

Orthodontic case analysis requires structured clinical reasoning in order to systematically gather, interpret and integrate extraoral, model-based and radiographic findings in order to make a diagnosis and develop a treatment decision. Undergraduate orthodontic education is delivered through a combination of lectures and clinical training, providing students with varied exposure to the discipline. However, these formats often offer limited opportunities for active engagement. Large-group lectures tend to position students as passive recipients, while clinical exposure frequently remains observational [1]. As a result, students may have insufficient opportunities to develop essential orthodontic competencies [2].

Web-based applications provide an opportunity to evaluate and improve the education sector. They facilitate evidence-based research on the adoption of digital education, leading to more interactive and intuitive e-learning experiences. Accessible at any time, these digital applications motivate learners and foster a more stimulating and engaging learning environment. Ultimately, this facilitates a broader shift from passive, teacher-centred learning towards active, learner-centred learning [3]. Despite growing evidence of their benefit, digital case-based learning tools are still underutilised in orthodontic education, and there is little empirical data on their effectiveness in clinical orthodontic diagnostic tasks [4, 5]. However, practical learning content plays an essential role in students' education, not only in orthodontics but across all dental disciplines. The further development of digital teaching, therefore, appears to be essential [6].

Among the digital tools developed to support orthodontic education [7–10], Orthotrainer is a web-based application specifically designed to facilitate the acquisition of orthodontic diagnostic competencies through structured, case-based practice [11]. Developed in close collaboration with the Audiovisual Media Center at RWTH Aachen University, the application is conceptually grounded in the Aachen Student Case Assessment Sheet, thereby ensuring alignment with established clinical teaching standards. Currently available to students and staff at RWTH Aachen University as part of a structured trial phase (version 2.1), Orthotrainer is anticipated to be released as a freely accessible resource for the wider dental education community in December 2026.

From a theoretical perspective, the design of the Orthotrainer is informed by principles of case-based learning and deliberate practice, both of which emphasise repeated exposure to authentic diagnostic tasks with structured feedback as a central mechanism for competence development [12, 13]. According to Cognitive Load Theory [14, 15], well-designed digital learning environments may support novice learners by reducing extraneous cognitive load through standardised workflows, and immediate corrective feedback, thereby freeing cognitive resources for schema acquisition [16]. Furthermore, Mayer's Cognitive Theory of Multimedia Learning [17, 18] suggests that learning is enhanced when information is presented through complementary visual and verbal channels, and when irrelevant material is minimised. Digital applications such as the Orthotrainer can operationalise these principles by combining interactive radiographic images, three-dimensional model visualisations, and structured diagnostic guidance within a single application, thereby promoting coherent mental model construction. At the same time, analogue case analysis may promote deeper spatial understanding and haptic engagement with physical models, which could differentially support specific diagnostic subdomains. These corresponding theoretical assumptions provide the rationale for the present study.

The aim of this study was to compare learning outcomes and clinical performance in orthodontic case analysis, comparing participants who used a purpose-built digital learning platform for case planning (Orthotrainer) with a control group that employed conventional analogue methods, including plaster models, printed diagnostic records, and manual measurement procedures. An analysis of the applicability and user experience of the learning methods was conducted as part of an orthodontic semester. Additionally, the corrective interventions employed by clinical staff for digital and analogue exams were evaluated. It is hypothesised that the web-based app Orthotrainer produces comparable learning outcomes to traditional teaching methods.

## Method

This study employed a controlled, parallel‑group design within the undergraduate orthodontic course. In parallel, a structured survey was conducted among experienced orthodontic educators to capture their perceptions and grading practices when assessing analogue versus digital examinations. This randomised controlled trial received approval from the Ethics Committee at RWTH Aachen University Hospital (EK 24/209). This study was reported in accordance with the CONSORT 2025 guidelines for randomised trials, and all participants provided written informed consent to participate in the study (Supp. Table 1). The data was collected during the winter semester between January 2025 and April 2025.

### Participants, eligibility criteria, and setting

Thirty dental students attending their second clinical orthodontic courses participated in this randomised controlled study at the medical faculty of RWTH Aachen University. The eligibility criteria included written informed consent and affiliation with a clinical orthodontic course. The exclusion criteria were (1) long-term absence from the course, (2) non-participation in both training sessions, (3) repetition of the course, and (4) absence of signed informed consent.

The curriculum for the second clinical year (semesters nine and ten) offers an in-depth examination of the essential elements of orthodontic diagnosis and treatment. All students received the same teaching methods and materials, ensuring consistent information transfer. In addition, all orthodontics lecturers from four universities (Aachen, Cologne, Düsseldorf and Berlin) were invited to take part in an anonymous cross-sectional survey examining the marking of paper-based examination papers in orthodontic training.

### Study design

All participants completed a baseline multiple-choice examination (MCE) to assess their orthodontic knowledge and a standardised questionnaire to collect students’ demographics (age, sex, semester) and readiness for online learning (Supp. Table 2 and 3).

An independent colleague carried out the randomisation of students, with no access granted to the principal investigators. Using central web-based computer-generated block randomisation, students were randomly assigned to either a digital group using the web-based learning app Orthotrainer or an analogue control group that received paper-based conventional orthodontic training. To guarantee students' full anonymity, an identifier was generated via a separate link, randomly created from responses to two individual questions and had to be used for the training sessions, the MCE, and the final module exam.

All students were given a step-by-step overview of the Orthotrainer through a ten-minute introductory video shared on the Moodle learning platform (RWTHmoodle, RWTH Aachen University, Aachen, Germany). Additionally, the video was shown in the lecture theatre prior to the first digital training.

Both groups completed two standardised training and assessment sessions (either digital or analogue) at a three-week interval within the regular orthodontic course and within the same predefined time frame, followed by a final module exam at the end of the semester. All students analysed identical patient cases according to the predefined study protocol. After each training session, the usability and user experience were evaluated using the System Usability Scale (SUS) [19] and the User Experience Questionnaire (UEQ) [20], as well as open-ended free-text feedback. The maximum time allowed for the training session was 90 min, and for the final exam, 150 min. The digital group only used the web-based learning app, Orthotrainer, during the scheduled training sessions. No free access to the programme was provided outside these sessions. The tasks, questions, and assessment criteria in training sessions were based on the diagnostic section of the final module exam. The sample solution was created by two experienced lecturers (maximum 54 points). Outcomes were evaluated using predefined scoring criteria, which were applied equally to both groups (Supp. Table 4). For the model analysis, the reference solution was generated using OnyxCeph[3]™ software (Image Instruments GmbH, Chemnitz, Germany). This software has previously been evaluated for digital orthodontic model analysis and treatment assessment and has been shown to provide valid and robust measurement outcomes [21–23]. Blinding of group affiliation for students and principal investigators was not feasible.

In addition, a structured questionnaire was distributed to 27 orthodontic educators at various universities to explore their current methods and perceptions regarding traditional exam grading in orthodontic education (Supp. Table 5).

### Baseline knowledge assessment: instrument development and validation

A purpose-developed MCE was used to assess baseline orthodontic knowledge prior to randomisation. The examination was designed to evaluate core principles of orthodontic diagnostics and was deliberately aligned in structure and format with the final summative assessment of the orthodontic curriculum, thereby ensuring both curricular relevance and comparability with established programme-level standards. Content validity was established through a two-stage process. First, all items were mapped to predefined learning objectives of the orthodontic curriculum to verify content representativeness. Second, the item pool was reviewed by three experienced orthodontic faculty members, who evaluated each item for clinical accuracy, instructional relevance, and alignment with the intended learning outcomes. Items were revised or eliminated based on reviewer consensus. An initial pool of 27 items was piloted with a cohort of 23 dental students from the preceding academic year who had completed the equivalent orthodontic curriculum, thereby minimising potential confounding factors arising from differences in course content. Item-level psychometric analysis was performed to identify items exerting a negative influence on overall scale reliability. Following iterative item reduction, a final instrument comprising 20 items was retained for use in the study.

Internal consistency of the final 20-item examination was assessed using Cronbach’s α in the pilot cohort [24]. The estimated coefficient was α = 0.794, 95% CI [0.626–0.896], exceeding the commonly accepted threshold of α ≥ 0.70 for acceptable internal consistency in medical education research [25]. The relatively wide confidence interval is consistent with the limited precision expected in a pilot sample of this size and supports cautious interpretation of the reliability estimate pending confirmation in larger independent samples [26].

For exploratory analyses, participants were classified into low- and high-performance subgroups based on a median split of baseline MCE scores. This approach reduces statistical power relative to continuous score modelling and should therefore be regarded as a heuristic stratification rather than a categorical distinction reflecting discrete competence levels [27].

### Digital group: web-based case planning using Orthotrainer

**Fig. 1:**
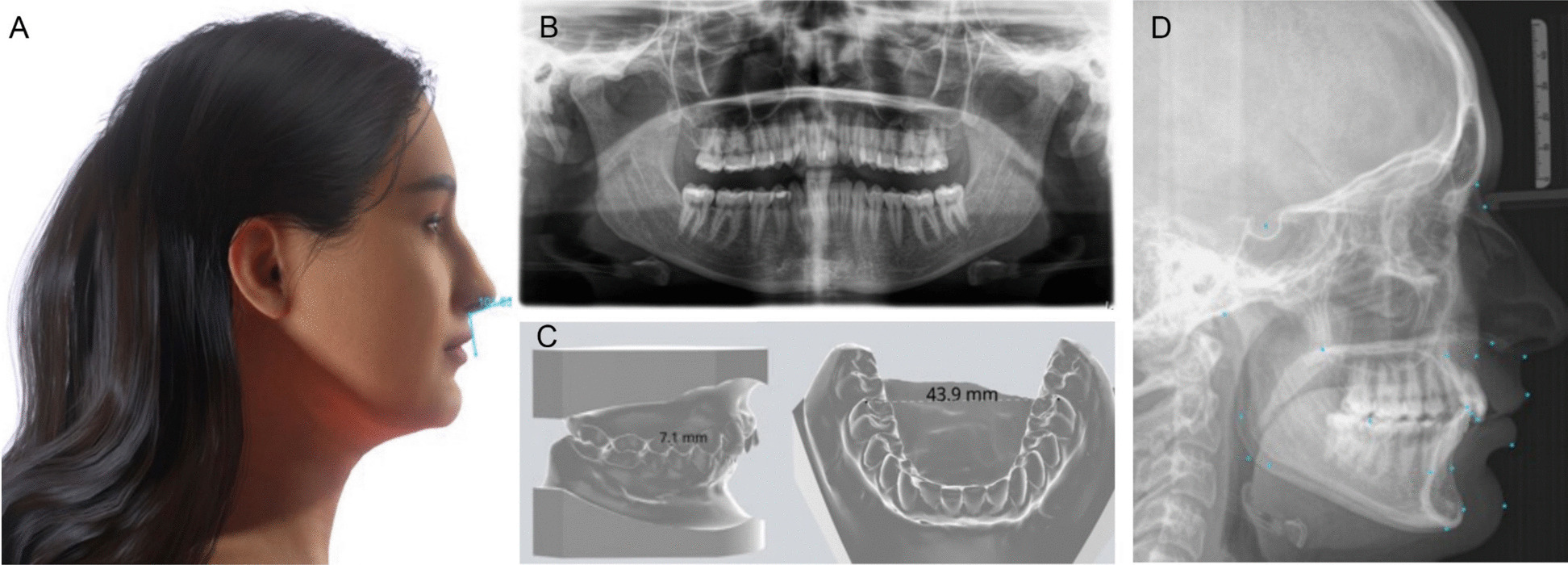
Illustration of the user interface of the e-learning application Orthotrainer. Extraoral profile image generated for profile analysis (**A**). The panoramic radiograph can be enlarged using a zoom function to facilitate detailed assessment (**B**). The 3D study model allows preset views, 360° rotation, and zoom functionality for model analysis (**C**). The lateral cephalometric radiograph can also be magnified using the zoom function to support landmark identification (**D**)

Participants in the digital group were provided with a standard desktop computer, mouse, and keyboard in a dedicated computer room. The digital records comprised digitised three-dimensional study models, an orthopantomogram, and a lateral cephalometric radiograph; an extraoral photo analysis was included as an extended diagnostic component. Animated dummies for extraoral analysis were created for both study groups using the Character Creator 4 software (Reallusion Inc., Taipei, Taiwan) and were used in both study groups (Fig. 1). Upon completion of each task, students received immediate corrective feedback in the form of sample solutions embedded within the application.

### Analogue group: conventional paper-based case-planning training

Students assigned to the analogue arm performed orthodontic case planning in a traditional lecture‑auditorium setting. Each learner received: a trimmed plaster model, a high-quality printed extraoral diagnostics, orthopantomogram, and a lateral cephalometric radiograph, as well as a calliper (Munich model, Dentaurum, Ispringen, Germany). The students used the set square and dividers as they saw fit. Measurements were recorded on the students' assessment sheet (Aachen student case assessment sheet). The students were given the solution to the case straight after the exercise.

### Usability and user experience measures

Usability and user experience were assessed using SUS [19] and UEQ [20]. To enable comparison with the analogue learning condition, the SUS items for the analogue group were adapted so that they referred to the traditional case-based assessment process rather than a digital tool. These adaptations were limited to changes in the wording of the context, while the original item structure and response scales were retained (Supp. Table 6).

### Final module examination

At the end of the semester, all participants completed the mandatory written final module examination required to pass the clinical orthodontics module, in accordance with inter‑university regulations. Each student received a trimmed plaster model, along with all the diagnostic records, printed in excellent quality and based on a real patient case. Students were required to perform a structured diagnostic analysis, and measurements were recorded on the students' assessment sheet (Aachen student case assessment sheet). In addition to the diagnostic tasks covered during the training sessions, the final examination included further diagnostic subcomponents as well as the formulation of a comprehensive treatment plan, thereby assessing both trained competencies and knowledge transfer.

### Orthodontics lecturers survey

An internally developed questionnaire was used to assess the demographic variables and years of teaching experience, as well as attitudes towards standardisation, correction workload and the potential role of digitalisation in examination processes. The questionnaire (Supp. Table 5) comprised both 5-point Likert scale items and open-ended questions. An invitation containing the survey link was sent by email to senior orthodontic lecturers at four German university sites (Aachen, Cologne, Düsseldorf and Berlin), asking them to forward it to eligible colleagues in their departments. Data were collected anonymously, and the final analysis included responses from 27 orthodontic educators.

### Outcomes

The primary outcomes included: (1) comparison of performance between the digital and analogue groups based on results from the training sessions and the module examination, and (2) evaluation of the applicability, acceptance, and user experience of both learning methods. Secondary outcomes comprised: (1) analysis of learning progress differences between low and high performing students, and (2) a descriptive evaluation of the Orthodontics lecturers survey on analogue examination correction practices. In terms of Kirkpatrick's hierarchy of educational outcomes [28], as adapted for health professions education [29, 30], the acceptance, applicability and user experience measures (SUS and UEQ) correspond to Level 1 (reaction); the students' online learning readiness and the educators' attitudes towards digitalisation and standardisation correspond to Level 2a (modification of attitudes and perceptions); and performance across the training sessions and the final module examination corresponds to Level 2b (acquisition of knowledge and skills).

### Sample size calculation

The sample size was not determined using an a priori power analysis, as the student cohort is a complete survey of the entire semester. All students who met the inclusion criteria were included. No a priori power analysis was conducted for the educator survey, as the analysis was descriptive in nature and did not involve inferential group comparisons. The final sample of *n* = 27 respondents represents a convenience sample of orthodontic educators from four university sites.

### Statistical analysis

All statistical analyses were conducted using R (version 4.4.0; R Foundation for Statistical Computing, Vienna, Austria) and JASP (version 0.19.1; JASP Team (2024)).

Potential differences between groups (analogue, digital) in baseline knowledge (MCE) and exam were assessed with independent-samples Welch’s t-tests.

A median split was performed based on the MCE scores to split the groups into low and high performers. Descriptive statistics (means, standard deviations, frequencies, and percentages) were computed for all items assessing participants' readiness to use online learning tools, using the *psych* package [31]. To examine changes in training performance (percentage of points achieved) over time (T1, T2) and differences between groups (analogue, digital), we conducted a series of linear mixed-effects models (*lme4*package[31],).

In a first model (Model 1), we examined the effects of time (T1, T2), group (analogue, digital), and their interaction on training performance. This model served as the primary analysis, testing whether performance changed across the two measurement points and whether this trajectory differed between the groups. In a second model (Model 2), we extended the primary analysis by including the diagnostic assessment types (extraoral -, model -, radiographic analysis) as a predictor. This allowed us to assess whether differences in performance between groups and across time points persisted after accounting for variability attributable to the diagnostic assessment types. In the third model (exploratory analysis, Model 3) we re-examined the effects of time and group on training performance while additionally differentiating participants within each group into low and high performers based on the median split of baseline knowledge assessment. This analysis was conducted to explore whether initial performance level moderated the trajectory of training success across groups. Given its exploratory nature, findings from this model should be interpreted with caution. All three models were estimated with restricted maximum likelihood (REML) and included random intercepts for participants. Statistical significance was evaluated at α = 0.05. Usability (SUS, UEQ) was analysed using linear mixed-effects models, considering the two training sessions (T1, T2) as within-subject factor, group (analogue, digital) as between-subject factor, and their interaction.

For all models, outliers were identified using cluster-level Cook’s distance. Influential participants were excluded in a sensitivity analysis. Further, non-significant interaction terms were removed from the final model to aid interpretability, following a stepwise model reduction approach. Where models included categorical predictors with more than two levels, results are described using estimated marginal means (EMMs) and pairwise contrasts rather than raw coefficients, as EMMs provide directly interpretable estimates independent of the reference category. Pairwise contrasts were Bonferroni-corrected for multiple comparisons. EMMs were computed using the *emmeans* package [32]. Full model coefficients are reported in the supplementary materials.

## Results

**Fig. 2:**
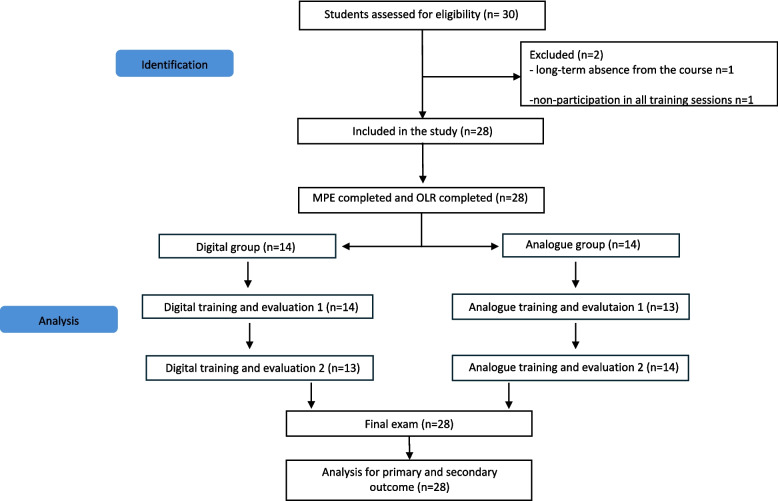
Study flowchart of patient cohort

A total of 28 students were included in the analyses, with 14 participants in each group. The sample comprised 15 female and 13 male students. Most participants were in the 9th semester (*n* = 18), while 10 students were in the 10th semester. The mean age was 26.0 years (SD = 4.23). Following randomisation, two students were excluded from the analyses because they did not attend the required training sessions (Fig. 2). Both groups completed the training sessions as planned within the predefined time limits and analysed identical patient cases according to the study protocol under standardised course conditions.

### Baseline knowledge assessment

Item analysis was conducted on response data from *N* = 30 participants across k = 20 dichotomously scored items. The mean total test score was M = 13.10 ± 2.68. Difficulty indices (*p*-values): Item difficulty indices ranged from *p* = 0.10 to *p* = 1.00 (M = 0.65). 11 of 20 items (55%) showed an acceptable difficulty level between 0.40 and 0.80. One item was classified as too difficult (*p* < 0.20). Three items fell in the borderline range between 0.20 and 0.40. Five items exceeded the ceiling threshold (*p* > 0.80, too easy). Discrimination indices (r_it): Corrected item-total correlations ranged from r_it = −0.06 to r_it = 0.54 (M = 0.16). Four items (20%) demonstrated good discrimination (r_it ≥ 0.30). Eight items fell below the minimum threshold of r_it < 0.20 and should be flagged for revision. Four items showed negative discrimination, indicating possible content or coding issues. Internal consistency: The internal consistency of the overall test was insufficient (< 0.60) (Cronbach's α = 0.56). No difference in the MCE scores, assessing the baseline knowledge in orthodontics, was found between the analogue group (M = 13.07 out of 20 points, ± 3.15, and the digital group (M = 12.86 out of 20 points, ± 2.41; t(24.35) = 0.20, *p* = 0.842, d = 0.08), so that the two groups can be assumed to have comparable starting conditions. Participants were categorised into low- and high-performance subgroups based on a median split of baseline MCE scores (cutoff = 13 points), resulting in an equal distribution: low performers (*n* = 6 analogue, *n* = 8 digital) and high performers (*n* = 6 analogue, *n* = 8 digital).

### Online learning readiness showed moderate to high digital readiness

**Fig. 3:**
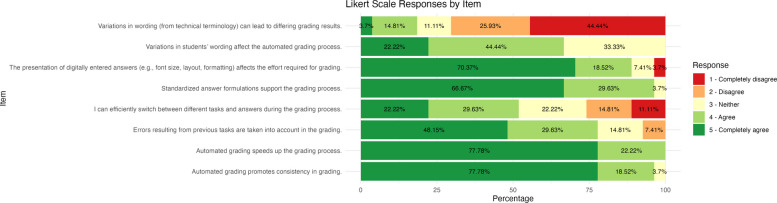
Online readiness assessment. Subjective assessment of the grading of paper-based case exams by lecturers (Aachen (*n* = 8), Cologne (*n* = 7), Düsseldorf (*n* = 4), and Berlin (*n* = 8)). The response options ranged from “Strongly agree” to “Strongly disagree”

Most students reported feeling confident about achieving the course objectives using digital materials (71.4%) and indicated that working with digital materials outside of traditional lectures was motivating (71.5%). In terms of learner autonomy, 60.7% of students stated that they could complete an online course without continuous support from an instructor. However, 66.1% indicated that direct contact with instructors remained important for their learning process. Although 51.8% found online learning outside the lecture hall more motivating than conventional courses, only 14.3% considered learning at home to be equivalent to learning in a classroom. Overall, the findings suggest that the students are moderately to highly digitally ready and have a sustained appreciation for teacher-led and face-to-face learning formats (Fig. 3).

### Comparable performance development over time in the training in both learning groups

**Fig. 4:**
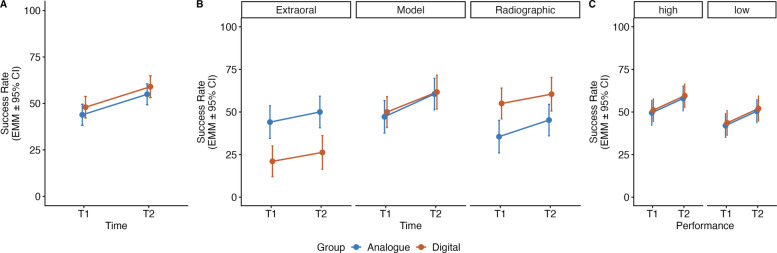
Learning performance in analogue and digital groups across training sessions. Data are presented as estimated marginal means (EMM) with 95% confidence intervals derived from linear mixed-effects models. **A** Overall success rates (%) across the two training sessions (T1 and T2), showing improvement over time in both groups. **B** Subdomain-specific performance for extraoral, model, and radiographic assessments. Group differences varied by domain, with higher performance in the analogue group for extraoral assessment and in the digital group for radiographic analysis, while model-based assessment showed improvement over time without clear group differences. **C** Subgroup analysis of performance by baseline competence (high vs low), indicating lower success rates during training of low performing students

In the primary model (Model 1), a significant main effect of time (T1, T2) on training performance was found, ß = 8.31, 95% CI [2.66, 13.96], t(50) = 2.96, *p* = 0.005, indicating an 8.31% increase in performance from T1 to T2 across groups (analogue, digital; Fig. 4A). There was no significant main effect of group (*p* > 0.05; Supp. Table 7, 8). Notably, one disproportionally influential participant was identified using cluster-level Cook’s distance and excluded in a sensitivity analysis; results were unchanged**.**

In Model 2, examination of the effect of assessment type on training performance similarly revealed an effect of time, that is an improvement from T1 to T2 across both learning groups, F(1, 130.64) = 12.29, *p* < 0.001. Further, a significant main effect of assessment type was revealed, F(2, 124.26) = 23.31, *p* < 0.001. The extraoral analysis scores were lower than model analysis scores (b = −19.5, 95% CI [−26.64, −12.39], t(124) = −6.65, *p* < 0.001) and lower than radiographic assessment (b = −13.7, 95% CI [−20.84, −6.59], t(124) = −4.67, *p* < 0.001). Model and radiographic assessment scores did not differ significantly (*p* > 0.05). Finally, a significant assessment type x group interaction was found, F(2, 124.26) = 24.52, *p* < 0.001 (Fig. 4B). The analogue group scored significantly higher than the digital group in the extraoral assessment (b = 23.37, 95% CI [7.31, 39.42], t(68.1) = 4.43, *p* < 0.001) and the digital group scored significantly higher than the analogue group in the radiographic assessment (b = −17.34, 95% CI [−33.40, −1.29], t(68.1) = −3.29, *p* = 0.002). No significant difference between groups was observed for the model assessment (*p* > 0.05). All remaining main effects and interactions were non-significant (*p* > 0.05; Supp. Table 9, 10).

Finally, in the exploratory Model 3, a time effect was revealed as well, ß = 8.46, 95% CI [2.82, 14.10], t(49) = 3.02, *p* = 0.004, with higher performance at T2. Further, a main effect of performance (high, low) was found, ß = −7.51, 95% CI [−14.85, −0.16], t(49) = −2.05, *p* = 0.045, with high performers showing a higher success rate than low performers. No effect of group (analogue, digital) was found, *p* > 0.05 (Fig. 4C; Supp. Table 11, 12).

#### Exam performance

**Fig. 5:**
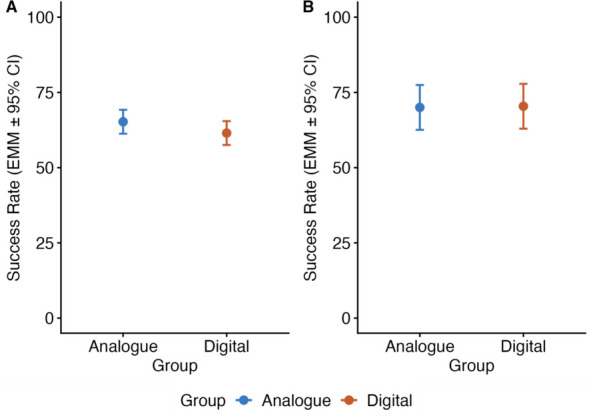
Transfer of knowledge to the final analogue module exam**.** Data are presented as estimated marginal means (EMM) with 95% confidence intervals. **A** Success rates (%) for the diagnostic components that were previously addressed during the training sessions, reflecting performance in familiar tasks. **B** Success rates (%) for additional components not explicitly covered during training, representing knowledge transfer

In the diagnostic results of the module exam relating to the training session, students achieved an average success rate of 63.38% (SD = 7.34), with no significant difference between the analogue and digital groups, t(26) = 1.37, *p* = 0.18, d = 0.52 (Fig. 5, Supp. Table 13). Exploratory analyses for knowledge transfer in the exam (further diagnostics and therapy only) showed no difference in the diagnostic and therapy success rate scores of the exam between the analogue group (M = 70.02%, SD = 17.04), and the digital group (M = 70.39%, SD = 8.81; t(19.48) = −0.07, *p* = 0.94, d = 0.03; Supp. Table 13).

### Usability evaluation of participants in both training sessions

**Fig. 6:**
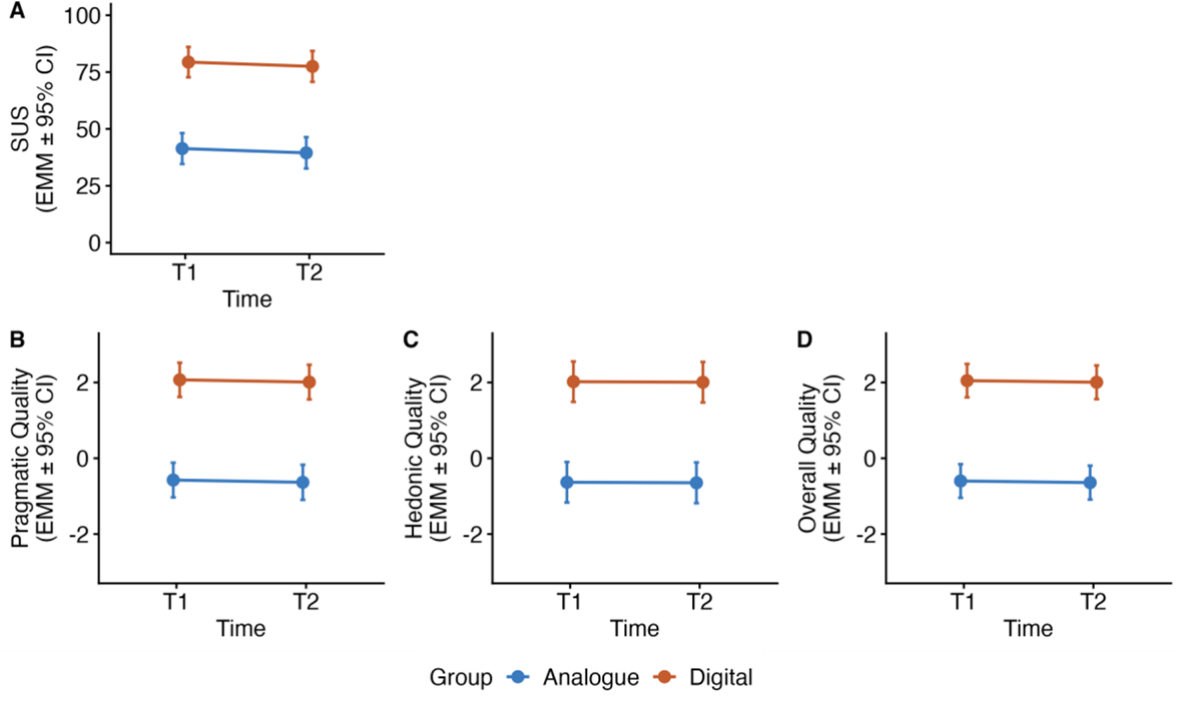
Usability and user experience outcomes across the training sessions for analogue and digital learning conditions. Data are presented as estimated marginal means (EMM) with 95% confidence intervals. **A** System Usability Scale (SUS) scores. **B** Pragmatic quality, (**C**) hedonic quality, and (**D**) overall quality as assessed by the User Experience Questionnaire (UEQ). Across all dimensions, higher ratings were observed for the digital condition, while no substantial changes over time were evident. Data are presented as estimated marginal means (EMM) with 95% confidence intervals

A significant main effect of group (analogue, digital) was revealed, ß = 38.02, 95% CI [29.27, 46.77], *p* < 0.001, showing higher SUS scores for the digital compared to the analogue application. That is, the mean SUS score for the digital application was 78.89 (SD = 14.11), indicating good usability of the application (Fig. 6A), and the usability score of the analogue application was rated lower with a mean score of 40.30 (SD = 11.19). There was no significant main effect of time (*p* > 0.05; Supp. Table 14, 15).

### User experience questionnaire in both training sessions

Across all users experience dimensions (pragmatic quality, hedonic quality and overall quality), significant differences between the analogue and digital conditions were observed (Fig. 6B, C, D). Pragmatic, hedonic and overall quality were always rated higher for the digital compared to the analogue application (pragmatic quality: β = 2.64, 95% CI [2.04, 3.25], *p* < 0.001; hedonic quality: β = 2.65, 95% CI [1.92, 3.39], *p* < 0.001; overall quality: β = 2.65, 95% CI [2.05, 3.25], *p* < 0.001). No significant main effect of time was observed across any of the dimensions, indicating that ratings did not significantly change between the first and second training session (*p* > 0.05; Supp. Table 16, 17). Sensitivity analyses identified two disproportionally influential participants in the pragmatic quality analysis based on cluster-level Cook’s distance; exclusion of these cases did not alter the results.

### Open feedback of the students

Open-ended feedback from students revealed different perceptions of analogue and digital approaches to case analysis.

The analogue approach was viewed positively due to the tactile and three-dimensional nature of physical models, as well as the ability to make direct annotations on models and paper-based records. However, students often described analogue workflows as time-consuming and error-prone, especially when it came to taking manual measurements with callipers. There were also concerns about the quality of printed radiographs, high paper consumption and limited opportunities for systematic feedback. Digital case analysis was evaluated largely positively. Students highlighted the intuitive usability and clear interface structure of digital tools, as well as the automated calculation of diagnostic parameters and the ability to compare results with reference values. They perceived measurements as faster and more efficient and considered radiographic visualisation to be clearer than printed images. Reported limitations mainly related to usability aspects, including marker size, the occasional requirement to scroll, and sporadic technical issues. Overall, students expressed a clear preference for further digital integration, including remote access to digital case analyses and hybrid teaching formats combining digital practice cases with face-to-face discussion.

### Educator survey on analogue exam correction

Data from 27 orthodontic educators were included in the analysis. Participants were recruited from four university sites (Aachen *n* = 9, Cologne *n* = 7, Düsseldorf *n* = 4, Berlin *n* = 8).

Teaching experience in orthodontic education varied, with five educators reporting less than one year of experience, twelve reporting 1–3 years, five reporting 4–6 years, and five reporting more than six years. Analogue exam correction was perceived as cognitively demanding and time intensive. Standardised assessment criteria and predefined answer formulations were consistently rated as facilitating efficiency and improving grading consistency. Free-text student responses were considered to allow interpretative variability. Handwritten responses by students were not perceived to influence grading outcomes; however, their readability was widely reported to increase correction workload. Overall correction effort and required concentration were rated as high. Open-ended responses highlighted a clear preference for increased digitalisation, improved standardisation, and more efficient correction workflows, including digitally supported or automated assessment approaches.

## Discussion

This exploratory randomised controlled trial compared digital, case-based training using the web-based Orthotrainer application with conventional analogue case analysis in undergraduate orthodontic education. The principal finding was that overall diagnostic performance did not differ between the two learning conditions, either during the training sessions or in the final module examination. This suggests that structured, web-based case analysis can support the acquisition of orthodontic diagnostic competencies to a comparable extent as traditional analogue methods.

During the data collection phase, the Orthotrainer was running version 2.1 and is currently available to students and staff at RWTH Aachen University for testing and feedback. This iterative approach enables user-based improvements and refinement of the application. Further continuous development and testing in a clinical setting with suitable study designs will provide the basis for its later implementation in the dental medicine curriculum.

In the present study, initial diagnostic knowledge was assessed using a curriculum-aligned MCE. There were no differences between the analogue and digital groups, indicating comparable initial knowledge conditions. However, the reliability of the instruments was found to be reduced in the baseline multiple-choice assessment. This may have impacted the accuracy of the baseline knowledge assessment and should be considered when interpreting comparisons of prior knowledge and subgroup analyses.

There was no difference in overall diagnostic performance between the digital and analogue groups in either the training sessions or the final examination. Both learning methods demonstrate performance improvements over time, which underscores the pedagogical validity of structured, case-based training regardless of the method used [11, 33, 34]. From the standpoint of deliberate practice theory [13], this result supports the idea that consistent, structured interaction with real diagnostic tasks, combined with feedback, fosters skill development regardless of whether the medium is digital or traditional. Both scenarios included key components: standardised case materials, clear diagnostic procedures, and sample solutions for self-assessment. While conventional formats may provide valuable hands-on experience, digital environments enable flexible, repeated, and self-directed engagement with complex cases, which may support the development of diagnostic reasoning skills.

Analogue case analysis was associated with better performance in extraoral analysis, while digital training was found to be more effective in radiological analysis. This finding has also been confirmed in the literature [7, 11]. Digital environments can improve landmark identification, measurement accuracy and visualisation in a lateral cephalometric radiograph. These domain-specific differences can be interpreted through the lens of Cognitive Load Theory and Multimedia Learning Theory. The digital environment may have reduced extraneous cognitive load in radiographic assessment by providing integrated zoom functions, automated measurement tools, and immediate visual feedback, features that align with Mayer's coherence and signalling principles [16, 17]. The reliability of different landmarks being identified varies, as identification is based on contrast with surrounding structures on the radiograph. Findings from extraoral profile analysis should be interpreted cautiously given the limited number of assessment points. Although the students work extensively with plaster models as part of the curriculum and are therefore more familiar with analogue workflows, the model analysis was carried out to the same high standard regardless of the group. Overall, these results imply that digital and analogue approaches are complementary rather than competing methods that support different aspects of diagnostic competence. Based on the MCE results, the influence of the learning modality appears independent of learners’ prior knowledge. Learners with low- and high-performance levels demonstrated similar improvements, with performance differences persisting throughout the training. This finding supports the hypothesis that students with a strong foundation in theoretical knowledge tend to perform better in case-based exercises [35, 36]. In the future, larger sample sizes could provide better insight into learning methods that depend on students’ prior knowledge.

The results of this usability study are consistent with previous evaluations of the Orthotrainer, which yielded a SUS score of 77.62 [11]. According to established benchmarks, this corresponds to a 'good' level of usability [37–39]. In the current study, the digital condition yielded a similar SUS score, highlighting the consistency of these results across groups.

In contrast, the analogue condition produced significantly lower SUS scores, indicating that traditional workflows are perceived as considerably less user-friendly. While this comparison should be interpreted with caution, given that the SUS was originally developed for digital systems [19, 39] and adapted for analogue use in this study, the magnitude of the observed difference indicates a clear advantage of digital learning environments in terms of user-friendliness. This superiority can be attributed to structured workflows, automated processes, and improved visualisation, all of which enable more intuitive interaction and reduce task complexity.

The UEQ results complement the SUS results, providing a more comprehensive assessment of the user experience [40]. In the digital method, UEQ scores consistently fell within the 'excellent' range across all dimensions.

This suggests that the positive assessment of the digital learning environment extends beyond functional usability to include pragmatic and hedonic quality aspects such as efficiency, stimulation, and perceived innovation. By contrast, analogue workflows are limited in terms of both usability and their ability to support these dimensions of the user experience.

Notably, the strong correlation between high SUS ratings and 'excellent' UEQ scores reinforces the reliability of these findings, indicating that the Orthotrainer is not only user-friendly but also provides an exceptionally positive overall user experience [41]. Taken together, these findings support the idea that digital learning tools can improve efficiency and learner engagement in complex educational settings, such as orthodontic training.

Students showed moderate to high willingness to engage with digital learning, which is consistent with previous literature [42, 43]. However, despite reporting confidence in using digital learning materials, they also emphasised the continuing importance of teacher guidance and face-to-face interaction. This was further supported by the open-ended feedback, in which students valued analogue workflows for tactile interaction and three-dimensional understanding, while identifying efficiency, automated calculations, improved visualisation, and instant feedback as key advantages of the digital method. Notably, students expressed a preference for hybrid learning formats combining digital case-based exercises with face-to-face tutor discussions, reflecting the broader trend in dental education towards blended learning [8, 44–47].

The reported usability limitations, including the size of markings and navigation, highlight that the user interface design can affect user experience and performance. This makes it essential to continually improve and develop the design based on scientific data and evaluation [46, 48]. In conclusion, personal guidance and feedback from lecturers cannot be replaced by digitisation [5, 49].

The educator survey provided additional insight into the context of analogue orthodontic case assessment. Analogue correction was consistently perceived as cognitively demanding and time-intensive, requiring sustained concentration. This highlights the resource implications of traditional assessment workflows. At the same time, however, educators emphasised that standardised grading criteria and predefined answer structures improve efficiency and grading consistency, thus supporting the importance of structured assessment design.

The interpretative variability associated with free-text student responses was identified as a further challenge, suggesting potential limitations in the reliability of analogue assessment formats. The strong preference expressed by educators for increased digitalisation and standardisation, as well as more efficient correction workflows, suggests that digital tools could support not only learning processes, but also assessment practices. From this perspective, digital orthodontic training platforms could help to improve the transparency, efficiency and scalability of case-based assessments in dental education.

### Limitations

Several limitations should be considered when interpreting the findings. The randomised controlled component of this study was conducted at a single centre with a relatively small sample size representing one academic semester in its entirety. Consequently, no a priori sample size calculation was performed, which may have limited the statistical power to detect smaller effects. The monocentric design also restricts the generalisability of the student-related findings. A further methodological limitation concerns the baseline knowledge assessment. Although the MCE demonstrated acceptable internal consistency in the pilot cohort (α = 0.794), the reliability in the study sample was considerably lower (α = 0.56), with several items showing poor or negative discrimination. This discrepancy may reflect differences in sample characteristics, cohort-specific response patterns, or instability of the reliability estimate due to small sample sizes. In contrast, the educator survey incorporated a multicentre sample across four university sites and was based on a priori sample size estimation, strengthening the external validity of the findings related to analogue assessment practices.

Additionally, the Orthotrainer was used in trial form, and any usability issues reported by participants may have influenced performance outcomes. Several potential sources of bias should also be considered. Firstly, the final examination was conducted exclusively in an analogue format, which may have introduced a methodological bias favouring students trained under conventional conditions. Furthermore, as students' prior training predominantly relied on analogue case analysis using plaster models, a familiarity bias may have been introduced, which could have affected model-based assessments. Finally, this evaluation addressed only the lower levels of Kirkpatrick's hierarchy: reaction (Level 1) and learning (Level 2), behavioural change in clinical practice (Level 3) and downstream benefits to patients or the wider organisation (Level 4) were not assessed and remain objectives for future longitudinal research.

## Conclusion

This exploratory randomised controlled trial suggests that digital, web-based training can complement conventional orthodontic education effectively. Digital training produced comparable diagnostic outcomes to analogue methods, demonstrating particular strengths in radiographic assessment. This indicates that structured digital environments may support specific domains of diagnostic competence and reasoning. Alongside the higher usability and user experience ratings observed for the digital format, these findings support the integration of web-based tools in blended learning concepts combining digital case-based practice with traditional face-to-face teaching in orthodontic education.

## Supplementary Information

Supplementary Material 1.

## Data Availability

The datasets used and/or analysed during the current study are available from the corresponding author upon reasonable request.
