# Supplementary material for: Utilising a web-based e-learning platform for orthodontic case analysis training: a randomised controlled trial
Source: BMC Med Educ. 2026 Jul 24;26:1205. doi: 10.1186/s12909-026-09968-0 (PMC13397713; doi:10.1186/s12909-026-09968-0)
Supplement: Supplementary file 1 — Supplementary Material 1. [file 12909_2026_9968_MOESM1_ESM.pdf]

Supplementary

Supplementary Table 1 Consort 2025 checklist for randomised trials

CONSORT 2025 checklist of information to include when reporting a randomised trial*

Section / Topic	No	CONSORT 2025 checklist item description	Reported on page no.
**Title and abstract**			
Title and structured abstract	1a	Identification as a randomised trial	1
	1b	Structured summary of the trial design, methods, results, and conclusions	1-2
**Open science**			
Trial registration	2	Name of trial registry, identifying number (with URL) and date of registration	Not applicable
Protocol and statistical analysis plan	3	Where the trial protocol and statistical analysis plan can be accessed	23
Data sharing	4	Where and how the individual de-identified participant data (including data dictionary), statistical code and any other materials can be accessed	23, Supplementary
Funding and conflicts of interest	5a	Sources of funding and other support (e.g., supply of drugs), and role of funders in the design, conduct, analysis and reporting of the trial	22
	5b	Financial and other conflicts of interest of the manuscript authors	22-23
**Introduction**			

Background and rationale	6	Scientific background and rationale	3-4
Objectives	7	Specific objectives related to benefits and harms	4-5
**Methods**			
Patient and public involvement	8	Details of patient or public involvement in the design, conduct and reporting of the trial	5
Trial design	9	Description of trial design including type of trial (e.g., parallel group, crossover), allocation ratio, and framework (e.g., superiority, equivalence, non-inferiority, exploratory)	5-6
Changes to trial protocol	10	Important changes to the trial after it commenced including any outcomes or analyses that were not prespecified, with reason	Not applicable
Trial setting	11	Settings (e.g., community, hospital) and locations (e.g., countries, sites) where the trial was conducted	5
Eligibility criteria	12a	Eligibility criteria for participants	5
	12b	If applicable, eligibility criteria for sites and for individuals delivering the interventions (e.g., surgeons, physiotherapists)	Not applicable
Intervention and comparator	13	Intervention and comparator with sufficient details to allow replication. If relevant, where additional materials describing the intervention and comparator (e.g., intervention manual) can be accessed	6-8
Outcomes	14	Pre-specified primary and secondary outcomes, including the specific measurement variable (e.g., systolic blood pressure), analysis metric (e.g., change from baseline, final value, time to event), method of aggregation (e.g., median, proportion), and time point for each outcome	9
Harms	15	How harms were defined and assessed (e.g., systematically, non-systematically)	Not applicable
Sample size	16a	How sample size was determined, including all assumptions supporting the sample size calculation	10
	16b	Explanation of any interim analyses and stopping guidelines	4
Randomisation:			

Sequence generation	17a	Who generated the random allocation sequence and the method used	6
	17b	Type of randomisation and details of any restriction (e.g., stratification, blocking and block size)	6
Allocation concealment mechanism	18	Mechanism used to implement the random allocation sequence (e.g., central computer/telephone; sequentially numbered, opaque, sealed containers), describing any steps to conceal the sequence until interventions were assigned	6
Implementation	19	Whether the personnel who enrolled and those who assigned participants to the interventions had access to the random allocation sequence	6
Blinding	20a	Who was blinded after assignment to interventions (e.g., participants, care providers, outcome assessors, data analysts)	6
	20b	If blinded, how blinding was achieved and description of the similarity of interventions	Not applicable
Statistical methods	21a	Statistical methods used to compare groups for primary and secondary outcomes, including harms	10-11
	21b	Definition of who is included in each analysis (e.g., all randomised participants), and in which group	10-11
	21c	How missing data were handled in the analysis	10
	21d	Methods for any additional analyses (e.g., subgroup and sensitivity analyses), distinguishing prespecified from post-hoc	11
**Results**			
Participant flow, including flow diagram	22a	For each group, the numbers of participants who were randomly assigned, received intended intervention, and were analysed for the primary outcome	Figure 1
	22b	For each group, losses and exclusions after randomisation, together with reasons	11
Recruitment	23a	Dates defining the periods of recruitment and follow-up for outcomes of benefits and harms	11
	23b	If relevant, why the trial ended or was stopped	Not applicable

Intervention and comparator delivery	24a	Intervention and comparator as they were actually administered (e.g., where appropriate, who delivered the intervention/comparator, how participants adhered, whether they were delivered as intended [fidelity])	10
	24b	Concomitant care received during the trial for each group	Not applicable
Baseline data	25	A table showing baseline demographic and clinical characteristics for each group	11
Numbers analysed, outcomes and estimation	26	For each primary and secondary outcome, by group:<br>● the number of participants included in the analysis<br>● the number of participants with available data at the outcome time point<br>● result for each group, and the estimated effect size and its precision (such as 95% confidence interval)<br>● for binary outcomes, presentation of both absolute and relative effect size	12-14
Harms	27	All harms or unintended events in each group	Not applicable
Ancillary analyses	28	Any other analyses performed, including subgroup and sensitivity analyses, distinguishing pre-specified from post-hoc	14
**Discussion**			
Interpretation	29	Interpretation consistent with results, balancing benefits and harms, and considering other relevant evidence	16-20
Limitations	30	Trial limitations, addressing sources of potential bias, imprecision, generalisability, and, if relevant, multiplicity of analyses	20-21

*We strongly recommend reading this statement in conjunction with the CONSORT 2025 Explanation and Elaboration and/or the CONSORT 2025 Expanded Checklist for important clarifications on all the items. We also recommend reading relevant CONSORT extensions. See www.consort-spirit.org

Supplementary Table 2 MCE to assess their orthodontic knowledge

1. Your identifier will be automatically entered here. If the field is empty, please enter the identifier yourself from the "My Data" section of the student portal.

2. Specify the FRS values in the following order: sagittal, vertical, dental.

- a. ANB, IOK-ML, base angle
- b. Jaw angle, WITS, IOK-IUK
- c. SNA, NL-NSL, IUK-NL
- d. SNB, ML-NL, IUK-ML

3. Which statement about standard deviation is most accurate?

- a. All values within the third standard deviation are classified as "tendential".
- b. All values within the second standard deviation are classified as "extreme".
- c. All values within the first standard deviation are classified as "mean".
- d. All values within the fourth standard deviation are classified as "manifest".

4. What is the best way to identify the "nasion" point in manual cephalometric evaluation?

- a. Anterior end of the frontonasal suture of the nasal bone (at the transition to the frontal bone)
- b. Posterior end of the nasomaxillary suture of the nasal bone (at the transition to the frontal bone)
- c. Posterior end of the frontonasal suture of the nasal bone (at the transition to the frontal bone)
- d. Anterior end of the nasomaxillary suture of the nasal bone (at the transition to the frontal bone)

5. How is the WITS value determined in manual cephalometric evaluation?

- a. Distance between A and B points on the occlusal plane
- b. Angle between point A, nasion and point B
- c. Distance between points A and B on the Nasio-Sella line
- d. Angle between point A, pogonion, point B

6. How is the Frankfurt Horizontal defined in manual cephalometric analysis?

- a. Connection of gonion and menton
- b. Connection of nasion and sella
- c. Connection of porion and orbitals
- d. Connection of point A and point B

7. Which method is used to determine the interincisal angle in manual evaluation?

- a. Angle between the tooth axes of the upper and lower incisors
- b. Angle between the tooth axis of the upper incisors and the Frankfurt horizontal
- c. Angle between the axis of the lower incisors and the mandibular plane
- d. Angle between the lower tooth axis and the occlusal plane

8. What angle is used to assess the mandibular position in profile analysis?

- a. SNB angle
- b. ANB angle
- c. Facial angle
- d. Nasolabial angle

9. Which teeth are included in the right support zone during the space analysis?

- a. 16, 15, 14
- b. 25, 24, 23
- c. 26, 25, 24
- d. 15, 14, 13

10. What tool is used to determine the symmetry of the dental arch during manual model analysis?

- a. Symmetrograph
- b. Bolton plate
- c. ruler
- d. Schmuth-Platte

11. What reference levels is the model analysis based on?

- a. Raphe-Median-Level
- b. Camper's plane
- c. Ear-eye level
- d. Hinge axis orbital plane

12. How is the posterior transverse width of the maxilla measured during manual model analysis?

- a. Distance between the palatal cusp tips of the second molars
- b. Distance between the approximal contacts of the premolars
- c. Distance between the tubercles of the canines
- d. Distance between the central fissures of the first molars

13. What occlusion at the 6-year molar describes the following occlusion?

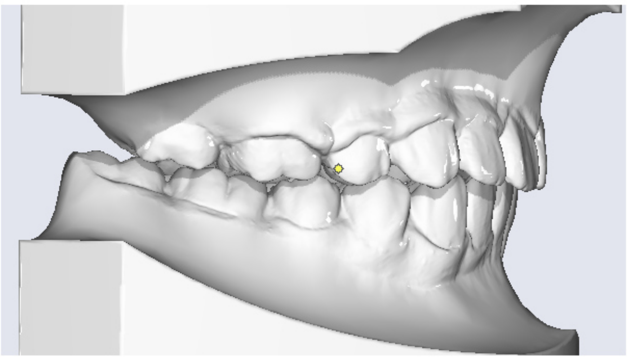

- a. NO
- b. 1 ½ PB DO
- c. 1 ¼ PB MO
- d. ½ PB DO

14. Which statement is least true about photo analysis?

- a. The labiomental angle and facial convexity can be measured en face.
- b. In the smiling picture, the center of the upper jaw can be determined to be the center of the face.
- c. In a gummy smile, the patient shows a lot of gingival exposure.
- d. The ears must be fully visible in the en face view.

15. How is the lower third of the face defined according to Kollmann?

- a. Subnasal – cutaneous gnathion
- b. Subnasal soft tissue pogonion
- c. Subnasal – soft tissue menton
- d. Subnasal – neck point

16. How is the facial convexity of the soft tissue profile measured in profile analysis?

- a. Nasion – Subnasal point – Menton
- b. Soft tissue nasion – subnasal point – soft tissue pogonion
- c. Soft tissue nasion – nasal tip – soft tissue menthon
- d. Nasion – Subnasal point – Gnathion

17. What can be observed on the following OPG?

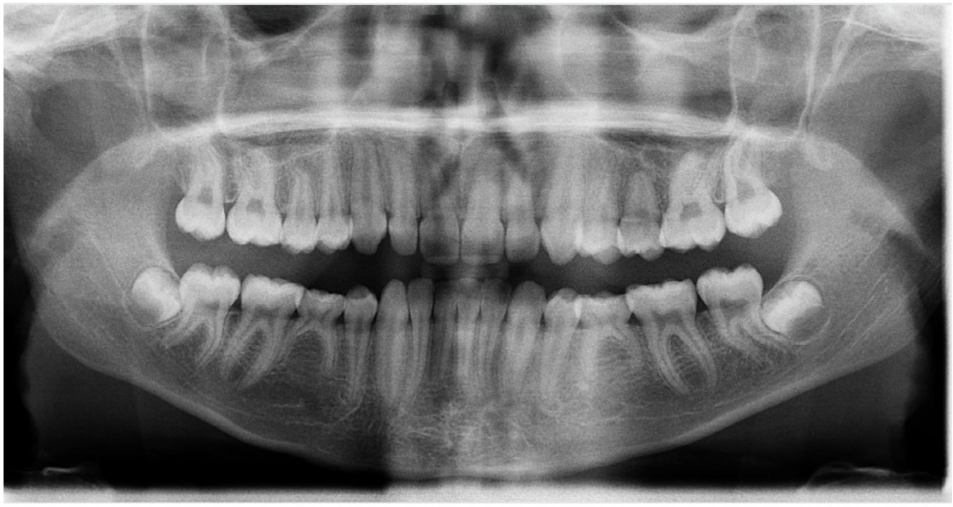

- a. Ankylosis
- b. persistence of primary teeth
- c. Extra teeth
- d. Schizodontia

18. What is the main function of an orthopantomogram (OPG) in orthodontics?

- a. Detailed representation of individual teeth
- b. Clear representation of the entire jaw and adjacent structures
- c. Measurement of skull size
- d. Analysis of temporomandibular joint movements

19. Which structures are typically visible on an OPG?

- a. Surpaorbital foramen
- b. hyoid bone
- c. Sella Turcica
- d. brain structures

20. Which statement about dentition disorders is most accurate?

- a. In hypoplasia, at least one tooth is missing.
- b. In oligodontia, at least 6 teeth per jaw are missing.
- c. Gemination is the process by which two germs fuse to form a tooth.
- d. Taurodontism can be classified into hypo-, meso-, and hyper-

21. Which additional X-ray image is often used in combination with an OPG in initial orthodontic diagnosis?

- a. Computed tomography (CT)
- b. Magnetic resonance imaging (MRI)
- c. Lateral cephalometric radiograph (CCR)
- d. Single-tooth film

Supplementary Table 3 Questionnaire to collect students' demographics (age, sex, semester) and readiness for online learning

Your identifier is automatically entered here. If the field is empty, please transfer the identifier from the "My Data" area in the study portal itself.

Which group were you assigned?

- ◯ Analogue
- ◯ Digital

Age in years

Enter only numerical values.

Gender:

- ◯ male
- ◯ female
- ◯ Divers

Semester

[Please select]

In the following, we ask you some general questions about digital teaching.

The following questions do not refer to the orthotrainer, but to your fundamental attitude towards digital teaching. Please estimate with each statement to what extent it applies to you.

	Does not apply at all		partly applies		fully true
Working with digital materials outside of the lecture motivates me.	◯	◯	◯	◯	◯
It is the same to study in the lecture hall or at home.	◯	◯	◯	◯	◯
I can achieve the learning objectives of this event with the help of digital materials.	◯	◯	◯	◯	◯
I have the feeling that I can improve the skills required in this event through online teaching to the same extent as through a lecture in the lecture hall.	◯	◯	◯	◯	◯
I find learning over the Internet outside the lecture hall more motivating than normal courses.	◯	◯	◯	◯	◯
I find that an online course can be taught completely without difficulties.	◯	◯	◯	◯	◯
I can complete a course completely over the Internet without the support of lecturers.	◯	◯	◯	◯	◯
I think the materials for online courses are better prepared than in the classic courses.	◯	◯	◯	◯	◯
As a student, I enjoy working with others or in groups.	◯	◯	◯	◯	◯
For me, direct contact with my lecturers is necessary to enable learning.	◯	◯	◯	◯	◯
I can discuss with other students outside of online classes.	◯	◯	◯	◯	◯
I can study well online in a group.	◯	◯	◯	◯	◯
I can network with other students outside of courses via the Internet.	◯	◯	◯	◯	◯

Supplementary Table 4 *Diagnostic* parameters and assessment criteria for training sessions 1 and 2

The diagnostic and assessment parameters are based on the final module examination of the orthodontic treatment course. A maximum of 54 points can be achieved in each training course.

Maximum: 54 points

Section	Measurement parameter	Allowed deviation[1]	Max points
	Extraoral profile analysis (max. 5 points)		
Facial profile	Soft-tissue angle N'–Sn–Pg' (N' = soft tissue nasion, Sn = subnasale, Pg' = soft tissue pogonion)	Angle is correct if: deviation < 1 SD	1.5
Nasolabial angle	Angle between nasal tangent and line Sn–Ls (Ls = labrale superius)	Angle is correct if: deviation < 1 SD	1.5
Face en face	Symmetry + facial thirds (Kollmann): Tr–N', N'–Sn, Sn–Gn' (Tr = trichion, Gn' = soft tissue gnathion)		1.0
Lip profile	Profile view: sagittal relation of upper vs lower lip		1.0
	OPG analysis (max. 4 points)		
All 32 teeth evaluated	Eruption status and dental findings: caries, fillings, root resorption		4.0 (32 × 0.125)
	Model analysis (max. 16 points)		
German KIG system	Determination of the KIG (Kieferorthopädische Indikationsgruppen = Orthodontic Indication Groups)		1.0
Dental arch form	Determination of the upper and lower dental arch form		2.0
Tooth shape and size	Recording of deviating tooth size and shape in relation to physiological anatomical norms		2.0

[1] Text answers are considered correct if they match the model solution.

Anterior and posterior arch widths maxilla	Distance between the cusps of canines and distance between the mesiobuccal cusp tips of first molars	Correct if: within ±1 mm from reference	4.0
Anterior and posterior arch widths mandible	Distance between the cusps of canines and distance between the the most gingival extension of buccal grooves of the first molar	Correct if: within ±1 mm from reference	4.0
Front tooth relation (12-22)	Vertical relation of the upper and lower front teeth to the occlusal plane		2.0
Curve of Spee	The measurement of the curve of Spee was performed on both sides and the mean of the measurements was calculated		1.0
	Space analysis (max. 11 points)		
Tooth sizes (15–25, 35–45)	Maximal mesiodistal width of the respective tooth (perpendicular to tooth axis and parallel to occlusal plane)	Correct if: within ±0,5 mm from reference	5.0 (20 × 0.25)
Required vs. available space	Right buccal segment (5–3), Left buccal segment (5–3), Anterior segment (2–2) in maxilla and mandible	Correct if: within ±1 mm from reference	6.0 (3 × 1) × 2
	Occlusion analysis (max. 4 points)		
Angle Class	Based on the relationship of the buccal groove of the mandibular first permanent molar and the mesiobuccal cusp of the maxillary first permanent molar		1.0
Sagittal occlusion	Static tooth pattern of the first molar and canine in the sagittal plane		1.0
Vertical discrepancies	Static tooth pattern of the first molars, premolars and canines in the vertical plane		1.0
Transverse deviations	Static tooth pattern of the first molars, premolars and canines in the transverse plane.		1.0
	Anterior tooth relationship (max. 2 points)		

Overbite	The maximum vertical overlap of incisors	Correct if: within ±1 mm from reference	1.0
Overjet	The maximum horizontal overlap of incisors	Correct if: within ±1 mm from reference	1.0
	Lateral cephalometric analysis (max. 12 points)		
Landmark placement	18 skeletal + 6 soft-tissue reference points placed on the lateral cephalogram	Correct if: ≤ 2 mm from reference	12.0 (24 × 0.5)

Supplementary Table 5 Orthodontics lecturers survey

1. University:

- ◯ Aachen
- ◯ Düsseldorf
- ◯ Cologne
- ◯ Berlin

2. How long have you been involved in orthodontic teaching?

- ◯ <1 year
- ◯ 1–3 years
- ◯ 4–6 years
- ◯ over 6 years

3. How often have you marked written exams in recent years?

- ◯ No prior experience with proofreading
- ◯ 1–2 corrections (entire cohort)
- ◯ 3–5 corrections (entire cohort)
- ◯ 6–10 corrections (entire cohort)
- ◯ More than 10 corrections (entire cohort)
- ◯ I regularly grade written exams (e.g., every semester).

4. In what setting do you primarily grade the exams?

- ◯ Alone
- ◯ In a team with other proofreaders

5. Please evaluate the following statements regarding the correction of written case evaluations in orthodontics:

	I disagree				agree
The legibility of handwritten exam answers affects the amount of correction required.	○	○	○	○	○
The handwriting of the exam participants influences the exam grade.	○	○	○	○	○
The way the students phrased their answers allows for different interpretations.	○	○	○	○	○
Standardized response formulations support the correction process.	○	○	○	○	○
Standardized evaluation criteria promote consistency in the evaluation.	○	○	○	○	○
Standardized evaluation criteria speed up the evaluation time.	○	○	○	○	○
Consequential errors from previous tasks are taken into account in the evaluation.	○	○	○	○	○
I can efficiently switch between different tasks and answers during grading.	○	○	○	○	○

6. Political questions:

	small amount		appropriate		high
How do you assess the duration of the correction process?	○	○	○	○	○
How high do you estimate the required level of concentration is during the correction process?	○	○	○	○	○

7. What measures would you like to see to make the correction process more efficient and fairer?

Supplementary Table 6 Analogue version of usability and user experience assessment

Answer the following questionnaire, assuming that you are performing an analog case evaluation.

Please estimate with each statement to what extent it applies to you.
1 = Strongly disagree
5 = Strongly agree

	1= Strongly disagree	2	3	4	5 = Strongly agree
I can very well imagine solving cases regularly with the help of analog models, pen and paper.	○	○	○	○	○
I find it unnecessarily complex to manually evaluate diagnostic documents analogously on paper.	○	○	○	○	○
I find it easy to manually evaluate diagnostic documents analogously on paper.	○	○	○	○	○
I think I would need support or help to be able to work on an analog case completely manually on paper.	○	○	○	○	○
I find that the different areas of responsibility are easy to carry out manually.	○	○	○	○	○
I find that there are too many inconsistencies in the analog case evaluation manually on paper.	○	○	○	○	○
I can imagine that most people quickly learn to master an analog case evaluation manually on paper.	○	○	○	○	○
I find the analog case evaluation manually on paper very cumbersome.	○	○	○	○	○
I felt very confident in the analog case analysis.	○	○	○	○	○
I had to learn a lot of things before I could manually work on an analog case.	○	○	○	○	○

Answer the following questionnaire, assuming that you are performing an analog case evaluation.

To evaluate the analog evaluation, please fill out the following questionnaire.
It consists of opposing pairs of properties that the system can have. Strats between the opposites are represented by circles. By ticking one of these circles, you can express your agreement to a term.

Please always tick an answer, even if you are unsure about the assessment of a pair of terms or find that it does not fit so well with the product. There is no "right" or "wrong" answer. Only your personal opinion counts!

Hindering	○	○	○	○	○	○	○	Supporting
Complicated	○	○	○	○	○	○	○	Simple
Inefficient	○	○	○	○	○	○	○	Efficient
Confusing	○	○	○	○	○	○	○	Clear
Boring	○	○	○	○	○	○	○	Exciting
Uninteresting	○	○	○	○	○	○	○	Interesting
Conventional	○	○	○	○	○	○	○	Original
Traditional	○	○	○	○	○	○	○	New

Supplementary Table 7: Linear mix-effects model to assess the effect of time (T1, T2) and group (analogue, digital) on performance in the training

Fixed Effects					
Parameter	Coefficient	SE	95 % CI	t(50)	p
(Intercept)	49.54	2.73	[44.06, 55.02]	18.16	< .001
Time	8.31	2.81	[2.66, 13.96]	2.96	0.005
Group [digital]	2.55	3.84	[-5.16, 10.25]	0.66	0.510
Random Effects					
Parameter	Coefficient	SE	95 % CI		
SD (Intercept: Participant)	6.97	2.31	[3.64, 13.34]		
SD (Residual)	10.30	1.42	[7.85, 13.50]		
Model Statistics					
Adjusted ICC	0.314				
Unadjusted ICC	0.280				
Conditional R2	0.388				
Marginal R2	0.108				

Supplementary Table 8: Descriptives of Training Data per group (analogue, digital) and time (T1, T2) (in Percentage)

Group	Time	n	Mean (SD)	Min	Max
Analogue	T1	13	43.74 (13.59)	22.73	70.00
Analogue	T2	14	55.34 (10.57)	36.14	71.82
Digital	T1	15	49.98 (9.92)	33.64	66.36
Digital	T2	13	53.90 (15.12)	10.91	67.95

Supplementary Table 9: Linear mix-effects model assesses the effect of time (T1, T2), group (analogue, digital), and assessment type (extraoral, model, radiographic) on performance in the training

Fixed Effects					
Parameter	Coefficient	SE	95 % CI	t(46)	p
(Intercept)	47.03	3.72	[39.67, 54.39]	12.63	< .001
Time	5.94	5.87	[-5.66, 17.55]	1.01	0.313
Group [digital]	-23.37	5.28	[-33.79, -12.94]	-4.43	< .001
Assessment Type [Model]	6.85	4.14	[-1.34, 15.03]	1.65	0.100
Assessment Type [Radiographic]	-6.64	4.14	[-14.82, 1.54]	-1.60	0.111
Assessment Type [Model] × Group [Digital]	25.34	5.87	[13.74, 36.94]	4.32	< .001
Assessment Type [Radiographic] × Group [Digital]	40.71	5.87	[29.10, 52.31]	6.93	< .001
Time × Group [Digital]	-0.70	8.36	[-17.23, 15.82]	-0.08	0.933
Assessment Type [Model] × Time	7.43	8.28	[-8.93, 23.79]	0.90	0.371
Assessment Type [Radiographic] × Time	3.79	8.28	[-12.57, 20.15]	0.46	0.648
(Assessment Type [Model] × Time) × Group [Digital]	-0.97	11.74	[-24.17, 22.24]	-0.08	0.935
(Assessment Type [Radiographic] × Time) × Group [Digital]	-3.57	11.74	[-26.77, 19.64]	-0.30	0.762
Random Effects					
Parameter	Coefficient	SE	95 % CI		
SD (Intercept: Participant)	8.57	1.87	[5.59, 13.14]		
SD (Residual)	15.20	0.96	[13.42, 17.22]		
Model Statistics					
Adjusted ICC	0.241				
Unadjusted ICC	0.158				
Conditional R2	0.501				
Marginal R2	0.343				

Supplementary Table 10: Descriptives (EMM) of Training Data per group (analogue, digital), time (T1, T2), and assessment type (extraoral, model, radiographic) (in Percentage)

Time	Group	Assessment Type	n	Mean	SD	Min	Max
T1	Analogue	Extraoral	13	44.23	27.30	0.00	75.00
T2	Analogue	Extraoral	14	50.00	19.61	25.00	75.00
T1	Digital	Extraoral	15	21.66	18.58	0.00	50.00
T2	Digital	Extraoral	12	25.00	21.32	0.00	75.00
T1	Analogue	Model	13	47.36	14.25	27.14	73.57
T2	Analogue	Model	14	60.56	13.15	37.85	86.42
T1	Digital	Model	15	50.61	11.27	35.00	67.14
T2	Digital	Model	12	60.41	9.37	40.00	75.00
T1	Analogue	Radiographic	13	35.69	18.36	31.25.	60.93
T2	Analogue	Radiographic	14	45.25	12.30	28.12	67.18
T1	Digital	Radiographic	15	55.62	20.86	20.31	93.75
T2	Digital	Radiographic	12	59.17	14.65	37.50	86.71

Supplementary Table 11: Exploratory linear mix-effects model to assess the effect of performance (high, low), group (analogue, digital), and time on success rate during training

Fixed Effects					
Parameter	Coefficient	SE	95 % CI	t(49)	p
(Intercept)	49.52	3.57	[42.35, 56.69]	13.87	< .001
Performance [low]	-7.51	3.65	[-14.85, -0.16]	-2.05	0.045
Group [digital]	1.56	3.65	[-5.78, 8.91]	0.43	0.671
Time	8.46	2.81	[2.82, 14.10]	3.02	0.004
Random Effects					
Parameter	Coefficient	SE	95 % CI		
SD (Intercept: Participant)	6.14	2.43	[2.82, 13.35]		
SD (Residual)	10.28	1.42	[7.84, 13.48]		
Model Statistics					
Adjusted ICC	0.263				
Unadjusted ICC	0.215				
Conditional R2	0.397				
Marginal R2	0.182				

Supplementary Table 12: Descriptives (EMM) of success rate in the training – per group (analogue, digital), performance (high, low), and time

Group	Performance	Time	n	M (SD)	Min	Max
Analogue	High	T1	6	49.52 (3.57)	42.25	56.79
Analogue	Low	T1	7	42.01 (3.41)	35.10	48.93
Digital	High	T1	9	51.08 (3.23)	44.50	57.67
Digital	low	T1	6	43.57 (3.57)	36.30	50.85
Analogue	High	T2	6	57.98 (3.58)	50.69	65.27
Analogue	Low	T2	6	50.47 (3.30)	43.78	57.17
Digital	High	T2	7	59.54 (3.37)	52.70	66.38
Digital	low	T2	8	52.04 (3.58)	44.74	59.33

Supplementary Table 13: Descriptives of Performance in Exam

Descriptives of exam performance without knowledge transfer (diagnostics and therapy) (in Percentage)

Group	n	Mean (SD)	Min	Max
Analogue	14	65.25 (7.23)	47.50	74.25
Digital	14	61.50 (7.22)	47.75	76.25

Descriptives of exam performance - knowledge transfer only (diagnostics and therapy) (in Percentage)

Group	n	Mean (SD)	Min	Max
Analogue	14	70.02 (17.04)	14.47	81.58
Digital	14	70.39 (8.81)	50.00	82.24

Supplementary Table 14: Linear mix-effects model to assess the effect of time (T1, T2) and group (analogue, digital) on SUS

Fixed Effects					
Parameter	Coefficient	SE	95 % CI	t(47)	p
(Intercept)	41.37	3.33	[34.67, 48.07]	12.42	< .001
Time	-1.89	2.57	[-7.07, 3.28]	-0.74	0.466
Group [digital]	38.02	4.35	[29.27, 46.77]	8.74	< .001
Random Effects					
Parameter	Coefficient	SE	95 % CI		
SD (Intercept: Participant)	9.30	2.09	[5.98, 14.46]		
SD (Residual)	9.09	1.33	[6.81, 12.11]		
Model Statistics					
Adjusted ICC	0.511				
Unadjusted ICC	0.161				
Conditional R2	0.846				
Marginal R2	0.686				

Supplementary Table 15: Descriptives (EMM) of SUS scores per group (analogue, digital) and time (T1, T2)

Group	Time	n	Mean (SD)	Min	Max
Analogue	T1	13	38.85 (9.72)	17.5	55.0
Analogue	T2	12	41.88 (12.84)	15.0	62.5
Digital	T1	14	81.25 (13.00)	57.5	100.0
Digital	T2	13	76.35 (15.33)	50.0	97.5

Supplementary Table 16:

Three linear mix-effects models to assess the effect of time (T1, T2) and group (analogue, digital) on pragmatic, hedonic and overall quality (UEQ) respectively

Pragmatic Quality (UEQ)

Fixed Effects					
Parameter	Coefficient	SE	95 % CI	t(47)	p
(Intercept)	-0.57	0.22	[-1.02, -0.12]	-2.56	0.014
Time	-0.06	0.15	[-0.36, 0.24]	-0.40	0.690
Group [digital]	2.64	0.30	[[2.04, 3.25]	8.85	< .001
Random Effects					
Parameter	Coefficient				
SD (Intercept: Participant)	0.68	0.13	[0.47, 1.00]		
SD (Residual)	0.53	0.08	[0.40, 0.70]		
Model Statistics					
Adjusted ICC	0.627				
Unadjusted ICC	0.186				
Conditional R2	0.890				
Marginal R2	0.704				

Hedonic Quality (UEQ)

Fixed Effects					
Parameter	Coefficient	SE	95 % CI	t(47)	p
(Intercept)	-0.63	0.26	[-1.16, -0.11]	-2.42	0.019
Time	-0.01	0.09	[-0.19, 0.16]	-0.16	0.870
Group [digital]	2.65	0.36	[1.92, 3.39]	7.30	< .001
Random Effects					
Parameter	Coefficient	SE	95 % CI		
SD (Intercept: Participant)	0.93	0.14	[0.70, 1.25]		

SD (Residual)	0.30	0.04	[0.23, 0.41]		
Model Statistics					
Adjusted ICC	0.904				
Unadjusted ICC	0.317				
Conditional R2	0.966				
Marginal R2	0.650				

Overall Quality (UEQ)

Fixed Effects					
Parameter	Coefficient	SE	95 % CI	t (47)	p
(Intercept)	-0.60	0.22	[-1.04, -0.16]	-2.76	0.008
Time	-0.04	0.11	[-0.26, 0.17]	-0.39	0.696
Group [digital]	2.65	0.30	[2.05, 3.25]	8.90	< .001
Random Effects					
Parameter	Coefficient	SE	95 % CI		
SD (Intercept: Participant)	0.73	0.12	[0.54, 1.01]		
SD (Residual)	0.37	0.06	[0.28, 0.50]		
Model Statistics					
Adjusted ICC	0.794				
Unadjusted ICC	0.219				
Conditional R2	0.943				
Marginal R2	0.724				

Supplementary Table 17:

Descriptives (EMM) of pragmatic, hedonic and overall quality (UEQ) per group (analogue, digital) and time (T1, T2) respectively

Pragmatic Quality (UEQ)

Group	Time	n	M (SD)	Min	Max
Analogue	T1	13	-0.50 (0.92)	-1.75	1.75
Analogue	T2	12	-0.73 (0.91)	-2.25	1.00
Digital	T1	14	2.02 (0.89)	0.50	3.00
Digital	T2	13	2.06 (0.78)	1.00	3.00

Hedonic Quality (UEQ)

Group	Time	n	M (SD)	Min	Max
Analogue	T1	13	-0.50 (0.92)	-2.5	3.25
Analogue	T2	12	-0.71 (0.97)	-2.5	0.50
Digital	T1	14	2.02 (0.95)	0.0	3.00
Digital	T2	13	1.94 (1.00)	0.0	3.00

Overall Quality (UEQ)

Group	Time	n	M (SD)	Min	Max
Analogue	T1	13	-0.50 (0.92)	-2.5	3.25
Analogue	T2	12	-0.71 (0.97)	-2.5	0.50
Digital	T1	14	2.02 (0.95)	0.0	3.00
Digital	T2	13	1.94 (1.00)	0.0	3.00
